# Inequity in level of healthcare utilization before and after universal health coverage reforms in China: evidence from household surveys in Sichuan Province

**DOI:** 10.1186/s12939-016-0385-x

**Published:** 2016-06-22

**Authors:** Hedda Flatø, Huafeng Zhang

**Affiliations:** Fafo Research Foundation, PO Box 2947, Tøyen, NO-0608 Oslo Norway

**Keywords:** Universal health coverage, Health equity, Health inequality, Health use, Access to healthcare

## Abstract

**Background:**

China has since the beginning of this millennium engaged in substantial Universal Health Coverage (UHC) reforms. This paper adds evidence on how equity in level of health service utilization changed after UHC reforms.

**Methods:**

Our study was based on household survey data from 30 counties in Sichuan province in 2004 and 2011. We introduce an unusual outcome variable, namely *level of healthcare utilization*. Concentration index (CI) was used to measure income based inequality in level of healthcare utilization. Horizontal index (HI) was used to assess whether inequalities are inequitable. We decomposed the concentration index to measure the factors contributing to inequality in level of utilization. Oaxaca type decomposition was applied to control whether identified changes were attributable to changed inequality or to other factors.

**Results:**

Pro-rich inequity in level of healthcare utilization increased after UHC reforms. Overall, a higher proportion of users sought services at county hospitals or higher in 2011 compared with 2004. Richer users were considerably more likely than the poor to seek care at hospitals rather than at clinics or health centers, and the pro-rich inequality in level of healthcare utilization was highly inequitable. Insurance enrollment became the main driver of pro-rich inequity in level of healthcare utilization after reforms, while health needs became less important for determining level of care, all disfavoring low income groups.

**Conclusions:**

Assessments of equity should pay attention to inequalities in level of healthcare utilization. Our results indicate that in China, wide insurance coverage is insufficient to ensure equity in level of healthcare utilization. On the contrary, type of insurance enrollment has become a main driver of inequity in level of utilization. Hence, equalizing health insurance schemes would be of crucial importance in order to improve health equity in China. Moreover, UHC reforms should strengthen the primary sector and limit non-needs based use of high-level hospitals in order to promote equitable use of healthcare services.

## Background

Universal Health Coverage (UHC) refers to securing “access to key promotive, preventive, curative and rehabilitative health interventions for all at an affordable cost, thereby achieving equity in access” [1]. A global UHC movement has emerged, firmly placing the issue on the global agenda. China has for more than a decade been engaged in health system reforms often seen as part of this movement [2–5].

Equity should be inextricably linked to UHC [6]. The World Health Organization has emphasized that “If universal coverage cannot be attained immediately, making progress fairly and equitably should be the main concern” [7]. But despite these intentions, recent literature has documented that UHC reforms in low –and middle-income countries may be followed by inequity in quality of care and in access to specialized services, with a pro-rich bias in utilization of higher-level services despite higher needs among poorer population groups [8]. In countries that have already achieved UHC, researchers have documented pro-rich bias in the use of curative specialist hospital services [9]. Yet equity of effective access is rarely included when measuring progress towards UHC [8].

China‘s government has introduced a number of policies and interventions aimed at achieving universal access to essential healthcare for all by 2020. The sheer size and scale of China’s health system reforms makes China an important case for studying the effects of UHC reforms on equity. Many low –and middle-income countries attempt to develop reforms in line with what has been done in China. For example, social health insurance schemes, which are at the core of China’s reforms, are important to many low –and-middle-income countries’ efforts to achieve UHC [10, 11].

China saw sharp increases in health inequalities based on income and geographical location after the introduction of market reforms in the early 1980s [12, 13]. Over the past decade China‘s government has introduced numerous measures to improve the healthcare system, including new insurance schemes as well as reforms targeting the primary sector, public hospitals, and pharmaceuticals. Building on initiatives dating back to the start of this millennium, the initial phase of the government’s health reform plan was to be achieved by 2011 [14].

New health insurance schemes covering huge and previously uninsured population groups are central to China’s UHC reforms. In 2003 more than half of China’s population was uninsured, but after reforms in 2011 more than 90 % of the population had some kind of insurance [15]. Although the insurance schemes achieved very wide coverage, the height and depth of coverage varies considerably across insurance schemes and localities [16, 17].

Insurance enrollment is mainly based on urban/rural residency and employment status. Urban workers employed in the public or private formal economy is the only population group that has enjoyed health insurance coverage for a long time. This group is covered by the tax financed Urban Workers Basic Medical Insurance scheme (UWBMI), which replaced an older scheme in 1997. The UWBMI is by far the most generous among the public insurance schemes.

Most rural residents had been uninsured since the dismantling of collective agricultural communes in the early 1980s. For rural residents a new insurance scheme has been introduced, called the New Rural Cooperative Medical Scheme (NCMS). The scheme targets all rural residents regardless of employment [18]. Local pilot projects started in 2003, and by 2005 the scheme was rolled out nation-wide. The NCMS is a shallow insurance scheme meant to provide risk-pooling for major illnesses and reducing the risk of rural dwellers falling into poverty due to illness.

For urban residents without formal employment, an insurance scheme called the Urban Residents Basic Medical Insurance (URBMI) was piloted from 2007. The scheme reached nationwide coverage in 2010. Targeting students, elderly, and unemployed urban residents not covered by the insurance scheme for urban workers, the URBMI is largely similar to NCMS in design.

Health service users in China can opt for health facilities at various levels in urban or rural localities. Facilities at different levels and in different localities vary widely with regard to service provision, staffing and quality of care [17, 19, 20]. The lowest level consists of village clinics in rural areas and street clinics in urban ones. They are staffed by one or a few doctors who often have limited education and deliver basic outpatient services including restricted medicine sales, consultations and some preventive services. At the second level, township or community health centers offer more advanced services and medicines, including in some cases simple surgery and inpatient services. The third level of health services consist of county-level hospitals, while hospitals at province and central level provide high-level specialized care. All hospitals offer both outpatient and inpatient care.

Crowding of higher-level hospitals and poor quality of lower-level facilities are recognized by policymakers as a major challenge for health reforms to address [21]. Exaggerated use of high-level hospitals is possible due to the fact that there is no referral system; hence patients are free to seek treatment at whichever type of hospital as long as they can pay, regardless of their medical needs. Moreover, fees for service and for-profit operation of hospitals provide managers and health workers with strong incentives to attract patients to high-level facilities and provide costly services, eroding physician ethics related to providing most appropriate care for patients’ needs [22]. Health system reforms from 2005 onwards included measures to strengthen public provision of primary and preventive care, including reduction of market incentives in lower level health facilities. However, no consensus was reached about the delivery of hospital services [23].

Health system reforms include some economic incentives for rural people to opt for lower-level facilities. In rural areas, NCMS typically only provides reimbursement for treatment at designated local facilities. And since 2009, public primary health facilities (clinics and health centers) have been required to stock and prescribe only drugs from a National Essential Medicine List and to sell them at zero profit, with higher insurance reimbursement than non-listed drugs [24]. Reforms also included payment reforms aimed at reducing for-profit motives for physicians at village clinics and township health centers. But a recent review concluded that still, “patients lack confidence in the quality of care provided by primary care providers and still bypass them to seek care at higher-level hospitals” [23]. At higher-level hospitals, pilots for reforms have “failed to yield useful lessons”, and profit incentives remained the main rule [23].

The question of how UHC reforms have affected equity in health service utilization in China has drawn increasing attention, but studies of the impact on equity in level of healthcare utilization are few and inconclusive. Nationwide analyses are mostly based on data from the China Health and Nutrition Surveys (CHNS). Assessments of inequality in healthcare utilization based on CHNS indicated that inequity in utilization had been reduced over time, although a strong pro-rich bias in inpatient care remained [25–29]. However, nationwide studies of other equity dimensions are less positive. Chen, Wu and Coyte’s assessment of children’s health achievement found that although the average health status of Chinese children had improved, health inequality among Chinese children had grown over the last two decades [30]. Utilization is closely related to affordability; Long et al. found that the affordability of healthcare for rural households with sick members had worsened despite the rapid increase of public funding to subsidize health insurance in China [31].

Other studies have examined the effects of specific UHC schemes on equity of utilization, mostly in selected counties. Wei Yang examined the effect of the New Rural Health Scheme on use of formal versus folk doctor care and concluded that the impact of the new insurance scheme on improving access to formal care for the poor was limited [27]. Yuan and colleagues found increased pro-rich inequity in inpatient care and pro-poor inequality in outpatient care among NCMS members in Junan County in 2011 compared with 2007 [32]. Liu et al.’s comparison of health insurance and equity in China and Vietnam found that in the 6 Chinese counties studied, health insurance membership had no significant impact on outpatient service utilization, while it was associated with higher utilization of inpatient services, especially for the higher income group [16]. A study of the impact of NCMS insurance benefit design on health care utilization in 2010 found that increased utilization of township and village-level outpatient care was experienced disproportionately by people who were poorer, while increased inpatient utilization overall and at the county level was experienced disproportionately by people who were richer [33]. As for urban insurance schemes, Zhou et al.’s study in Shaanxi province concluded that both the urban workers’ scheme (UWBMI) and the urban residents’ scheme (URBMI) reduced pro-rich inequity of inpatient utilization, but for outpatient utilization the scheme for workers (UWBMI) increased pro-rich inequity whereas the scheme for other urban residents (URBMI) increased pro-poor inequity [34].

This paper adds evidence from household surveys in 30 counties in Sichuan province in 2004 and 2011. The paper aims to measure to what extent inequity in *level of health service utilization* changed after introduction of UHC reforms. Furthermore, it aims to identify determinants of such inequity. We adopt a more nuanced measure of level of utilization than what is common in the literature, since our data allows for analyzing inequalities in the *type* of facility used rather than crude inpatient/outpatient categories. In line with Kutzin’s recommendations for assessing health system performance, our unit of analysis is the population and health system in the survey area as a whole, rather than being concerned only with specific schemes or population groups [10].

## Methods

The data utilized in this study are from two surveys conducted in 2004 and 2011. The most recent survey covered 30 counties in Sichuan province affected by the 2008 Sichuan earthquake. Interviews were completed with 3,841 households. 2004 data come from the survey Measuring Living Conditions in Western China (MEDOW) [35]. 3079 households were interviewed in the 30 counties in Sichuan province studied in this paper. Both surveys were organized by Chinese Academy of Science and Technology for Development (CASTED) with technical support from the Norwegian research foundation Fafo.

Fundamentally the healthcare system and insurance schemes in the survey area is similar to the rest of China. The 30 selected counties include urban areas in and around the capital Chengdu, rural areas in the fertile Sichuan plains, and remote mountain villages. The sample is predominantly rural: In 2004 only 10.6 % of household members (50 persons) had urban household registration, while in the 2011 sample about 22.9 % (452 persons) held urban residence registration (Table 1).

**Table 1:** Descriptive statistics

Variable	2004	2011
Level of healthcare utilization (used high-level services during past 30 days)^a^	144 (30.6 %)	920 (46.7 %)
Mean annual housheold income per capita (Yuan)	3245	8211
Mean Ln annual housheold income per capita	7.8	8.6
Chronic sickness or disability	257 (54.6 %)	1154 (58.5 %)
Acute sickness past 30 days	249 (52.9 %)	1127 (57.2 %)
Gender (women versus men)	258 (54.8 %)	1100 (55.8 %)
Mean age	50.0	45.9
Urban versus rural residency	50 (10.6 %)	452 (22.9 %)
Marital status		
Single, never married	33 (7.0 %)	126 (6.4 %)
Married	376 (79.8 %)	1645 (83.5 %)
Divorced/separated	62 (13.2 %)	200 (10.1 %)
Educational level		
No school or incomplete primary	251 (53.3 %)	361 (18.3 %)
Primary	122 (25.9 %)	778 (39.5 %)
Junior secondary	85 (18.0 %)	543 (27.5 %)
Senior secondary or higher	13 (2.8 %)	289 (14.7 %)
Employment		
Non-agricultural work	46 (9.8 %)	455 (23.1 %)
Agricultural work	268 (56.9 %)	731 (37.1 %)
Odd work	50 (10.6 %)	177 (9.0 %)
Retired	60 (12.7 %)	297 (15.1 %)
Not in labor force	47 (10.0 %)	311 (15.8 %)
Insurance type		
Insurance from work unit (2004)/Public or urban workers insurance (2011)	34 (7.2 %)	163 (8.3 %)
Social insurance (2004)/Urban residents insurance (2011)	4 (0.8 %)	124 (6.3 %)
Insurance from village (2004)/New rural cooperative medical insurance (2011)	22 (4.7 %)	1523 (77.3 %)
Other insurance	5 (1.1 %)	170 (8.6 %)
No insurance	406 (86.2 %)	97 (4.9 %)
Sample size	471	1971

^a^Use of county or higher-level hospital = 1, use of township/community health center or village/street clinic = 0

The 30 counties had to varying degrees seen destruction due to the earthquake in 2008. By 2011 reconstruction in the counties was largely completed; 82 % of households reported that their living conditions were the same as or better than before the earthquake [36]. Although the earthquake injury rate was high in absolute numbers, the proportion of sampled household members reported to have been injured in 2008 was only 1.4 %. A qualitative investigation of access to healthcare in one of the affected villages found that distribution systems and medical insurance systems were not subject to long-term alteration as a consequence of the earthquake [36]. Many health facilities were rebuilt or repaired during the 2 years following the earthquake, hence an unusually high number of facilities in the surveyed counties may have been new or recently repaired. County governments have flexibility to determine the specific design of insurance schemes and health service distribution systems, which thus may be different in other localities.

This study examines the type of facilities visited by household members who had used healthcare services during the past 30 days. We will refer to this dependent variable as “Level of healthcare utilization”. As explained above, in the Chinese context there is a substantial qualitative difference in the characteristics of clinics and health centers, on the one hand; and hospitals, on the other. We therefore constructed a dichotomous variable by which use of lower-level facilities = 0 and higher-level facilities = 1. Lower-level healthcare facilities include facilities referred to in the health reform plan as “basic” or “grassroots”: Village or street clinics; township health centers or community health centers. Higher-level facilities are defined as public hospitals at county level or higher. Specifically, we measured the highest level of facilities used; hence, individuals who had used both high and low-level services during the past 30 days were considered to be “high-level users” and coded 1. This happened only in 17 cases. In the analysis, only household members aged 15 or older who had used any of these facilities, and who had complete data on all independent variables, were included. This amounted to a sample size of 1971 individuals in 2011, and 471 individuals in 2004.

Independent variables were classified into four components, in accordance with Concentration Index decomposition methodology [37–39]: (1) individual income, (2) need variables (3) other explanatory variables, and (4) the residual term. The income variable used is the per capita natural log of annual household income. Household income is measured by self-reported wages, pensions and unemployment benefits from all household members in addition to household agricultural income, non-agricultural family business income, and household subsidies. Need variables include self-reported acute illness during the past 4 weeks; and self-reported chronic disease. Other variables include health insurance; household location (urban or rural); gender and age; marital status; educational level; and occupation. Insurance includes enrollment in New Rural Cooperative Health Scheme (NCMS); enrollment in Urban Residents Basic Medical Insurance (URBMI) and enrollment in Urban Workers Basic Medical Insurance (UWBMI).

Following Wagstaff and others [37–41], this paper employs concentration index and decomposition of the concentration index to measure the degree of inequity in level of health service utilization, and to estimate how much income, health insurance and other variables contributed to or reduced inequality in 2004 and 2011. The concentration curve plots the cumulative proportion of level of healthcare utilization against the cumulative proportion of the sample, ranked by income. If there is no income related inequality, the concentration curve is diagonal. The concentration index is defined as twice the area between the concentration curve and the diagonal. The concentration index is bounded between −1 and 1. If there is no inequality, the concentration index is zero. If there are higher utilization rates among the rich, the concentration curve is pushed below the diagonal and the concentration index takes on a positive value; if utilization is higher among the poor, the concentration index takes on a negative value and the concentration curve is pushed above the diagonal. The formula for the concentration index can be written as follows [38]:

1 $$ C=\frac{2}{n\mu }{\displaystyle {\sum}_{i=1}^n{y}_i{R}_i-1} $$

Where *C* is the concentration index, *y*_*i*_ is level of healthcare utilization for the *i*th person, μ is the mean of *y*, and *R*_*i*_ is the fractional rank of the *i*th person in the income distribution.

Decomposition of the concentration index allows for assessing whether inequalities identified by the concentration index amounts to inequities, by taking needs variables into account. As proposed by Wagstaff and others, the concentration index can be decomposed into four components: (1) individual income, (2) need variables, such as chronic and acute sickness, (3) other explanatory variables, including health insurance, educational level, employment, etc., and (4) the residual term, reflecting inequality that cannot be explained by systematic variation across income groups in the other variables [40]. A straightforward way of decomposing the contributions of explanatory factors in the context of a linear additive explanatory model, such as:

2 $$ {y}_i = \propto + {\displaystyle {\sum}_k{\beta}_k{x}_{ki}+\kern0.75em {\varepsilon}_i} $$

Where ∝, *β*_*k*_ are coefficients, *x*_ki_ is k_th_ regressor for individual i, and Ɛ is the disturbance term.

The concentration index can then be written as [40]:

3 $$ \mathrm{C} = {\displaystyle {\sum}_k\left({\beta}_k{\overline{x}}_k\ /\mu \right){C}_k+G{C}_{\varepsilon }\ /\mu } $$

Where *C*_*k*_ is the concentration indexes for contributing variables *x*_*k*_ (defined analogously to *C*), ${\overline{x}}_k$ is the mean of x_k_,and *GC*_*ε*_ is the generalized concentration index of Ɛ_i_, defined as $G{C}_{\varepsilon } = \frac{2}{n}{{\displaystyle {\sum}_{i=1}^n{\varepsilon}_iR}}_i$.

The concentration index is made up of two components: A deterministic component and a residual component. The deterministic component is equal to a weighted sum of the concentration indices of the k regressors, where the weight or “share” for x_k_ is the elasticity of y with respect to x_k_ (evaluated at the sample mean). The residual component term reflects inequality which cannot be explained by systematic variation across income groups in the x_k_.

In order to assess to what extent inequalities identified by the concentration index amounts to inequity, a horizontal inequity index (HI) can be computed to analyze to what extent individuals with equal values on the need component obtain the same treatment. HI can be computed by subtracting the contribution of need variables (CN) from the concentration index of health service utilization (CM) [41]. A linear approximation is given as:

4 $$ {y}_i={\displaystyle {\sum}_k{\beta}_k^m{x}_i^k+\kern0.75em {u}_i} $$

Where the β_k_^m^ are the partial effects of each independent variable and evaluated at sample means, $\mathrm{dy}/\mathrm{d}{\overline{\mathrm{x}}}_{\mathrm{k}}$; and u_i_ is the implied error term which includes approximation errors. The approximation is linearly additive, and the decomposition approach can be applied.

In order to analyze the influence of determinants on visits to higher-level or lower-level healthcare facilities, a non-linear logistic regression model for binary responses has to be used to decompose the concentration index. The two-step approach to neutralizing non-need variables, and the decomposition approach, does not hold for non-linear models. As suggested by Doorslaer and others, ‘partial effects’ can be used in non-linear models to approximate the decomposition approach in linear models [42]. The concentration index for y in a non-linear model can be written as:

5 $$ \mathrm{C}={\displaystyle {\sum}_{\mathrm{k}}\left({\upbeta}_{\mathrm{k}}^{\mathrm{m}}{\overline{\mathrm{x}}}_{\mathrm{i}}^{\mathrm{k}}/\upmu \right){\mathrm{C}}_{\mathrm{k}} + {\mathrm{GC}}_{\mathrm{u}}/\upmu} $$

The modified version of the decomposition approach for non-linear models provides an estimate of the contributions of individual factors to the overall inequality in level of healthcare utilization. Although easier to conduct, it is noted that due to linear approximation error, the HI estimate is not unique, and not in general same as the estimate from the indirect standardization with nonlinear models (2).

There are various methods to decompose change in the concentration index. Wagstaff and others suggested to take the difference of all the components of the decomposition, so as to unravel the effect of each components on the changes of healthcare utilization inequalities [40].

6 $$ \varDelta C={\displaystyle {\sum}_k\left({\beta}_{kt}{\overline{x}}_i^{kt}/{\mu}_t\right){C}_{kt} - {\displaystyle {\sum}_k\left({\beta}_{kt-1}{\overline{x}}_i^{kt-1}/{\mu}_{t-1}\right){C}_{kt-1} + \varDelta \left(G{C}_{ut}/{\mu}_t\right)}} $$

Oaxaca-type decomposition can be applied to decompose the factors attributable to changes of inequalities or changes of their elasticities on the determinants of healthcare services utilization [43].

7 $$ \varDelta C={{\displaystyle {\sum}_k\upeta}}_{kt}\left({C}_{kt} - {C}_{kt-1}\right) + {\displaystyle {\sum}_k{C}_{kt-1}\ \left({\upeta}_{kt} - {\upeta}_{kt-1}\right) + \varDelta \left(G{C}_{ut}/{\mu}_t\right)} $$

Or

8 $$ \varDelta C={\displaystyle {\sum_k}_{kt-1}\left({C}_{kt} - {C}_{kt-1}\right) + {\displaystyle {\sum}_k{C}_{kt}\ \left({\upeta}_{kt} - {\upeta}_{kt-1}\right) + \varDelta \left(G{C}_{ut}/{\mu}_t\right)}} $$

Where η_*kt*_ and η_*kt* − 1_ are elasticities of concentration indices, calculated as ${\beta}_k{\overline{x}}_k\ /\mu$.

## Results

Among household members age 15+ included in the survey, about 23 % of the total population had used some kind of services both in 2011 and in 2004. Only those who had used healthcare services at public hospitals, health centers or clinics during the past 30 days were included in the analysis. A higher proportion of users visited higher-level facilities in 2011 compared with 2004. In 2011, 47 % of users sought care at county level hospital or higher, whereas 53 % used health centers or clinics. By contrast, in 2004, 31 % sought care at county level hospital or higher, whereas 69 % used health centers or clinics. In total 1971 individuals who visited the types of health facilities studied in this paper had complete data for all the independent variables in 2011. For 2004 altogether 471 individuals had visited the types of low-level and high-level health facilities studied here. Table 1 shows descriptive statistics for all variables.

Table 2 shows the Concentration index (CI) and horizontal index (HI) for utilization and need variables in 2004 and 2011. CI was positive in both 2004 and 2011, indicating pro-rich inequality in level of healthcare utilization. In order to assess whether the inequality in level of use was inequitable, need must be taken into account. Horizontal inequity indexes, whereby the contribution of needs variables were deducted from the CI, was calculated to assess equity in level of healthcare utilization. In both 2004 and 2011, needs variables (including chronic disease and acute illness) had negative contributions on inequality in level of utilization: HIs were almost 20 % higher than CIs. From 2004 to 2011, both CI and HI in level of healthcare use increased slightly by 7–8 %.

**Table 2:** Concentration index (CI) and horizontal index (HI) for level of health service utilization

	Level of healthcare utilization	
	CI	HI
2004	0.065096	0.076880
2011	0.069624	0.08289
Change	0.004528	0.00601

The concentration index for level of healthcare utilization was decomposed based on logistic regression. Regression results for 2004 and 2011 are displayed in Table 3. In 2011 those holding new rural cooperative medical insurance were less likely to opt for high-level healthcare and more likely to seek low-level care compared with those who had other types of insurance. By contrast, in 2004 health insurance had no significant impact on level of healthcare utilization. Chronic sickness status had a strong effect on level of healthcare utilization whereas acute sickness did not have significant effect. People with higher education and those with non-agricultural work were more likely to select higher-level healthcare services in both years. Urban residency had a statistically significant positive effect in 2004, but not in 2011. Age, gender and marital status had insignificant effects on level of utilization.

**Table 3:** Logistic regression result 2004 and 2011, partial effect dy/dx

	Level of healthcare utilization			
	2004		2011	
	dy/dx	z	dy/dx	z
Ln annual housheold income per capita	−0,013	−0,43	−0,017	−1,20
Chronic sickness or disability	0,083	1,58	0,130	4.28^c^
Acute sickness past 30 days	0,052	1,19	−0,008	−0,35
Gender (women versus men)	−0,049	−1,00	0,012	0,39
Age	0,007	0,71	0,003	0,44
Age square	−0,009	−0,86	−0,006	−1,16
Urban versus rural	0,184	2.34^b^	−0,029	−0,70
Marital status (Divorced/separated)				
Single, never married	−0,057	−0,36	0,102	1,17
Married	0,034	0,36	0,080	1,48
Educational level (Senior secondary or higher)				
No school or incomplete primary	−0,348	−2.43^b^	−0,142	−2.17^b^
Primary	−0,259	−1.77^a^	−0,134	−2.48^b^
Junior secondary	−0,145	−0,98	−0,095	−1.90^a^
Employment (non-agricultural work)				
Agricultural work	−0,213	−2.66^c^	−0,165	−3.79^c^
Odd work	−0,075	−0,82	−0,122	−2.27^b^
Retired	0,041	0,33	−0,071	−1,19
Not in labor force	0,047	0,45	−0,036	−0,76
Insurance type (Insurance from village/New rural cooperative)				
Insurance from work unit (2004)/Public or urban workers insurance (2011)	0,640	2.95***	0,504	6.47^c^
Social insurance (2004)/Urban residents insurance (2011)	0,000	0.00	0,236	3.60^c^
Other insurance	0,282	1,03	0,164	3.46^c^
No insurance	0,469	2.61^c^	0,139	2.12^b^
N	471		1971	
chi2	71,69		188,06	

^a^Significance at the 10 % level

^b^Idem., 5 %

^c^Idem., 1 %

Table 4 shows the decomposition results and the contribution of each variable to inequalities in level of utilization, based on equation 5. Each variable’s contribution to the concentration index equals the concentration index for explanatory variable x_k_, weighted by the elasticity of that variable’s concentration index. Based on equation 3, the elasticity of each variable’s concentration index was calculated as ${\beta}_k{\overline{x}}_k\ /\mu$. Results are displayed in the columns entitled “elasticities”. The columns entitled “Concentration indices” show income based inequality in each explanatory variable. In order to calculate each variable’s contribution to the concentration index for level of healthcare utilization, we multiply the concentration index for each variable by its elasticity. The contribution to inequality in level of healthcare utilization for each explanatory variable is displayed in the columns entitled “Contributions to overall CI”. If the contribution to the concentration index for level of health utilization is positive, the variable contributed to pro-rich inequality, and vice versa.

**Table 4:** Decomposition of inequality in level of healthcare utilization

	Elasticities		Concentration indices		Contributions to overall CI		
	2004	2011	2004	2011	2004	2011	Change
Income variable					−0.017	−0.022	−0.005
Ln annual housheold income per capita	−0.325	−0.33	0.053	0.066	−0.017	−0.022	−0.005
Demand variables					−0.012	−0.013	−0.001
Chronic sickness or disability	0.147^a^	0.168^c^	−0.06	−0.082	−0.009	−0.014	−0.005
Acute sickness past 30 days	0.092	−0.011	−0.032	−0.051	−0.003	0.001	0.004
Other variables					0.077	0.117	0.04
Gender (women versus men)	−0.071	0.012	0.031	−0.007	−0.002	−0.000	0.002
Age	1.153	0.288	−0.018	−0.023	−0.021	−0.007	0.015
Age square	−0.758	−0.412	−0.044	−0.042	0.033	0.017	−0.016
Urban versus rural	0.063^b^	−0.015	0.264	0.311	0.017	−0.005	−0.021
Marital status					0.005	0.002	−0.003
Single, never married	−0.012	0.014	−0.189	−0.021	0.002	−0.000	−0.003
Married	0.088	0.149	0.026	0.013	0.002	0.002	−0.000
Educational level					0.034	0.018	−0.016
No school or incomplete primary	−0.600^b^	−0.054^b^	−0.098	−0.248	0.059	0.013	−0.045
Primary	−0.219^a^	−0.116^c^	0.003	−0.056	−0.001	0.007	0.007
Junior secondary	−0.085	−0.063^a^	0.287	0.034	−0.024	−0.002	0.022
Employment					−0.004	0.027	0.031
Agricultural work	−0.392^c^	−0.137^c^	−0.001	−0.193	0.000	0.026	0.026
Odd work	−0.026	−0.024^b^	0.061	−0.055	−0.002	0.001	0.003
Retired	0.017	−0.022	−0.147	0.077	−0.002	−0.002	0.001
Not in labor force	0.015	−0.012	−0.033	−0.085	−0.000	0.001	0.002
Insurance type					0.016	0.064	0.047
Insurance from work unit (2004)/Public or urban workers insurance (2011)	0.076^c^	0.081^c^	0.193	0.643	0.015	0.052	0.037
Social insurance (2004)/Urban residents insurance (2011)	0.000	0.032^c^	0.803	0.168	0.000	0.005	0.005
Other insurance	0.009	0.034^c^	0.457	0.187	0.004	0.006	0.002
No insurance	1.370^c^	0.015^b^	−0.002	0.014	−0.002	0.000	0.003
Residual term					0.017	−0.012	−0.029

^a^Significance at the 10 % level

^b^Idem., 5 %

^c^Idem., 1 %

The base categories for explanatory variables are divorced/separated, senior secondary or higher education, non-agricultural work, Insurance from village/new rural cooperative

In 2011, insurance type became the dominant contributor to inequality in level of healthcare utilization (CI contribution 0.064). In 2004, insurance was the second-largest contributor (CI contribution 0.016). From the concentration indices for insurance variables, we see that richer people were considerably more likely to be enrolled in urban workers’ medical insurance (UWBMI) rather than insurance for rural residents (NCMS). There were small and insignificant income differences between the holders of rural insurance (NCMS) and those who had urban residents insurance (URBMI) or other insurance. When multiplying the insurance concentration indices with their elasticities, we see that insurance type overall contributed positively to inequality in level of healthcare utilization in both survey years, and that the effect increased strongly from 0.016 in 2004 to 0.064 in 2011. Most of the contribution of insurance to inequality in level of healthcare utilization came from having urban workers’ insurance rather than rural insurance. Apart from insurance, the contributions of several other explanatory variables to inequality in level of healthcare utilization changed substantially between 2004 and 2011. Employment status also increased its contribution greatly, from −0.004 in 2004 to 0.027 in 2011. In 2004, the largest contribution to inequality in level of healthcare use came from inequality in educational level (CI contribution 0.034). The contribution of educational level decreased almost by half by 2011.

The negative concentration indices for needs variables chronic/acute sickness show that poor people were more likely to have poor health, particularly chronic sickness. When multiplying the needs variables’ CIs with their elasticities, we see negative contributions to inequality in level of healthcare utilization for both 2004 and 2011, with little change across time. The marginal effect of income was insignificant both years. Residual variables contributed strongly to pro-poor inequality in level of healthcare use in 2004, while the sign of the residual variables’ contribution was negative in 2011.

The decomposition of the concentration index in Table 4 does not provide information on to what extent changes in explanatory variable’s contributions to inequality in level of healthcare utilization can be attributed to changes in elasticities rather than to change in inequality. Oaxaca decomposition was conducted to assess to what extent the changes in CI contributions were due to changes in elasticities or to changes in inequality of the variables. Table 5 displays results for two types of Oaxaca decomposition. The first two columns display results based on equation 7, whereas the third and fourth column show results based on equation 8. Regardless of decomposition method, change in the decomposed concentration index between years 2004 and 2011 is the same. Decomposed change from year 2004 to 2011 is displayed in the final column in the table.

**Table 5:** Oaxaca-type decomposition of change for inequality in level of healthcare utilization, 2004-2011

	Elasticities 2011*(Ckt-Ckt-1)	Ckt-1*(Elasticities 2011-Elasticities 2004)	Elasticities 2004*(Ckt-Ckt-1)	Ckt*(Elasticities 2011-Elasticities 2004)	Decomposed Change
Ln annual housheold income per capita	−0.004	0.000	−0.004	0.000	−0.005
Chronic sickness or disability	−0.004	−0.001	−0.003	−0.002	−0.005
Acute sickness past 30 days	0.000	0.003	−0.002	0.005	0.004
Gender (women versus men)	0.000	0.003	0.003	−0.001	0.002
Age	−0.001	0.016	−0.005	0.020	0.015
Age square	−0.001	−0.015	−0.001	−0.015	−0.016
Urban versus rural	−0.001	−0.020	0.003	−0.024	−0.021
Marital status	0.000	−0.003	−0.003	0.000	−0.003
Single, never married	0.002	−0.005	−0.002	−0.001	−0.003
Married	−0.002	0.002	−0.001	0.001	0.000
Educational level	0.031	−0.047	0.124	−0.140	−0.016
No school or incomplete primary	0.008	−0.053	0.090	−0.135	−0.045
Primary	0.007	0.000	0.013	−0.006	0.007
Junior secondary	0.016	0.006	0.022	0.001	0.022
Employment	0.025	0.006	0.081	−0.050	0.031
Agricultural work	0.026	0.000	0.075	−0.049	0.026
Odd work	0.003	0.000	0.003	0.000	0.003
Retired	−0.005	0.006	0.004	−0.003	0.001
Not in labor force	0.001	0.001	−0.001	0.002	0.002
Insurance type	0.007	0.040	0.053	−0.006	0.047
Public or urban workers insurance	0.036	0.001	0.034	0.003	0.037
Urban residents insurance	−0.020	0.026	0.000	0.005	0.005
Other insurance	−0.009	0.011	−0.002	0.005	0.002
No insurance	0.000	0.002	0.022	−0.019	0.003
Total	0.052	−0.019	0.246	−0.213	0.033

The base categories for explanatory variables are divorced/separated, senior secondary or higher education, non-agricultural work, Insurance from village/new rural cooperative

For insurance type and employment status, changes in inequality rather than in elasticities are dominant in their positive contribution to inequality in level of healthcare utilization. Educational level had higher changes in elasticities than inequalities; the inequality of educational level had increased quite much but was offset by the changing elasticities. Urban/rural residency also had higher changes in elasticities than in inequalities, with only very little change in inequality of residency. For need variables, changing inequalities and changing elasticities had reinforcing effects, both contributing to decreasing inequality.

## Discussion

Overall utilization rates had not changed significantly in 2011 compared with the situation before UHC reforms, in 2004. For both years, around 23 % of the population had used some kind of healthcare services during the past 30 days. However, a higher proportion of users visited higher-level facilities in 2011 compared with 2004 (47 % of users in 2011 compared with 31 % in 2004). This runs contrary to the reforms’ aims of shifting utilization more towards primary care.

Our results indicate that it was mainly the rich who enjoyed the increase in high-level healthcare utilization. Positive CIs for 2004 and 2011 indicate that a higher proportion of rich health service users had opted for county-level hospitals or higher while a higher proportion of the poor sought lower-level services at village/street clinics or community health centers (Table 2). Controlling for inequality in health needs, HIs were almost 20 % higher than CIs both years, meaning that differences between rich and poor were even larger when needs were taken into account. This demonstrates that distribution of high-level versus low-level healthcare was inequitable: Inequalities between income groups was not based on differences in needs but rather based on differences in income. Despite the massive expansion of health insurance and other health reforms that had happened between 2004 and 2011, both inequality and inequity in level of health service use had increased slightly.

Logit regression (Table 3) showed that in 2011, rural medical insurance (NCMS) enrollees were disadvantaged in using high-level healthcare compared with persons enrolled in the urban workers’ basic medical insurance (UWBMI) or the urban residents’ basic medical insurance (URBMI). In 2004, by contrast, health insurance was not significantly important in determining the level of healthcare utilization. Chronic sickness status, higher educational level, and formal non-agricultural employment were also important and significant determinants of level of healthcare utilization.

Decomposition of the concentration index found that the contribution of need variables to inequality in level of healthcare utilization was negative in both 2004 and 2011, indicating that the poor had greater need of high-level healthcare services. Yet, the contributions of need variables became less important for explaining inequality in level of health services used. Instead, the contribution of other variables to inequality in level was strong and increased from 2004 to 2011.

The contribution of insurance type and employment status to inequality in level of healthcare utilization increased dramatically after health insurance reforms. As demonstrated in Table 4, the contribution of employment status and insurance to the CI for level of healthcare utilization increased from very low or negative in 2004 to very high and positive in 2011, disfavoring poor people. It should be noted that during the same time period, insurance coverage increased dramatically in the counties covered by our survey, particularly for rural residents (see Table 1). By 2011 94 % of our sample had some kind of health insurance, whereas only 12 % had health insurance in 2004. Coverage of insurance schemes catering to rural residents increased from 2.7 % in 2004 to 77 % in 2011. The coverage of insurance targeted at urban residents without formal employment had also increased, from 1.3 % of our sample in 2004 to 6.6 % in 2011, whereas insurance for urban workers had been stable at around 7 % both years.

The dramatic increase in inequality caused by insurance type and employment status was to some extent offset by reductions in the contributions from education level, need and income variables. Residual variables contributed a lot to pro-poor inequality in level of health service use in 2004, while the sign of the residual variables’ contribution was opposite in 2011, suggesting that there remains a good deal of unexplained variation in changes in inequity beyond the variables examined in this analysis.

Oaxaca decomposition (Table 5) showed that the higher pro-rich contributions of insurance type and employment status in 2011 compared with 2004 were caused by higher inequality rather than changing elasticities. This indicates that what had changed after UHC reforms was inequality in access to insurance type – which was again closely related to employment status. Hence, changed distribution of insurance type was the key mechanism behind inequality in level of healthcare utilization after UHC reforms in China.

## Conclusions

Equity of access to healthcare services is a central goal in the global universal health coverage (UHC) movement, and it was one of the aims for China’s UHC reforms over the past decade. Our analysis of 30 counties in Sichuan province indicates that reforms significantly increased pro-rich inequity in the level of healthcare services obtained by users. Affluent people were even more likely to opt for high-level facilities after reforms than before, whereas it was mainly the poor – with more medical needs - who resorted to using lower-level facilities. Variation in type of insurance enrollment became the main driver of income based inequality in level of healthcare utilization after reforms.

Our study adds evidence to a small body of research on how UHC reforms may reinforce or even exacerbate inequity in health. It highlights the need for assessments of progress towards UHC to include equity measures. It shows that measuring progress on health equity by studying only inequalities in overall use or outpatient/inpatient use may conceal inequities in the level of healthcare obtained. Specifically, our results provide an argument for including measures of inequality in the characteristics of health services or facilities used.

Under the current administration in China yet another cycle of health reforms has been introduced, according to Hsiao and Yip (2015) implying less emphasis on equity and more reliance on privatization and the hospital sector [23]. Hsiao and Yip [23] predict that China’s health system will become a two-tiered system, with high-level hospitals largely serving affluent households. Our results indicate that such a development was already underway by the end of the first phase of health system reforms. Political action is needed if this trend is to be reversed.

Our results demonstrate that if Chinese authorities do wish to prioritize equity in health, the impressive strides taken towards universal health coverage are not sufficient. Indeed, by the end of the first reform phase, differences in insurance schemes seemed to exacerbate inequality in level of healthcare utilization in China. Therefore, equalization of insurance schemes is crucial for further reforms to contribute to equity. Moreover, our results indicate that although reforms have included steps towards strengthening the primary sector, stronger measures are needed in order for people to opt for clinics and health centers not only because they cannot afford to go elsewhere, but also because they have confidence that such facilities can provide appropriate care for their needs. Finally, while reforms hitherto do include some positive measures aimed at inducing more use of lower-level services – such as economic incentives and quality improvement – few steps have been taken to prohibit unnecessary use of high-level care. Screening and referral systems can be developed in order for access to higher-level services to be determined by patients’ needs rather than by their ability to pay.
